# Self-Expanding Stent-Assisted Reconstruction of Distal Supra-Aortic Vessels in Acute Type A Aortic Dissection: A Comparative Analysis

**DOI:** 10.3390/jcm15218243

**Published:** 2026-10-23

**Authors:** Bowen Li, Feng Shi, Zhipeng Hu, Zongli Ren, Zhiyong Wu, Luocheng Li, Zhiwei Wang

**Affiliations:** Department of Cardiovascular Surgery, Renmin Hospital of Wuhan University, Wuhan 430060, China; bowenli@whu.edu.cn (B.L.); shifeng@whu.edu.cn (F.S.); huzhipengphd@whu.edu.cn (Z.H.); renzongli0723@gmail.com (Z.R.); wzy2008@whu.edu.cn (Z.W.)

**Keywords:** aortic dissection, supra-aortic vessels reconstruction, self-expanding stent, neuroprotection, hybrid surgery

## Abstract

**Background**: Supra-aortic vessel reconstruction during total aortic arch replacement in acute Stanford type A aortic dissection patients with dissected distal supra-aortic vessels remains technically challenging; proximal supra-aortic vessel replacement alone carries a high risk of neurological complications secondary to intimal inversion. This study aims to evaluate clinical outcomes of self-expanding stent-assisted supra-aortic vessel reconstruction compared with proximal replacement of supra-aortic vessels alone. **Methods and Results**: We performed a single-center retrospective comparative analysis of 49 consecutive patients undergoing emergency total aortic arch replacement for acute Stanford type A aortic dissection from January 2022 to December 2023. Patients were divided into two groups according to intraoperative anatomy and surgeon decision: self-expanding stent-assisted supra-aortic vessel reconstruction (SAVR, *n* = 26) and proximal replacement of supra-aortic vessels alone (PRVA, *n* = 23). Neurological outcomes related to supra-aortic vessel intimal inversion, operative parameters, blood product transfusion, and short-term complications were analyzed. The mean follow-up was 18.2 ± 4.1 months (range, 12–24 months). The SAVR group exhibited significantly lower supra-aortic vessel stenosis rate (3.8% vs. 30.4%, *p* < 0.001) and zero cases of intimal inversion-related luminal collapse versus five cases in the PRVA group (0 vs. 5, *p* = 0.02). Numerically fewer cases of symptomatic stroke (0% vs. 8.7%, *p* = 0.09) and radiographic cerebral infarction (3.8% vs. 13.0%, *p* = 0.17) were observed in the SAVR group, though the differences did not achieve statistical significance. Red blood cell and fresh frozen plasma transfusion requirements, mechanical ventilation duration, and ICU stay length were significantly lower in the SAVR cohort. Cardiopulmonary bypass time and 30-day mortality were comparable between groups. **Conclusions**: Self-expanding stent-assisted supra-aortic vessel reconstruction represents a promising technical option during total arch replacement for acute Stanford type A aortic dissection with distal supra-aortic dissection, associated with superior branch vessel patency and favorable numerical trends in neurological and recovery-related endpoints, with a comparable early-term safety profile. Potential benefits require further validation in larger cohorts.

## 1. Introduction

Acute Stanford type A aortic dissection (ATAAD) represents one of the most life-threatening cardiovascular emergencies. Without surgical intervention, the overall mortality reaches approximately 23.7% within the first 48 h, with an incremental risk of 1–2% per hour; although this estimate is lower than early reports from the 1950s, the associated risk remains substantial [1,2]. Contemporary surgical strategies have evolved from conservative hemiarch replacement toward more comprehensive total aortic arch replacement, driven by evidence supporting improved long-term outcomes and reduced reintervention rates [3].

The major technical challenge of supra-aortic vessel reconstruction during total aortic arch replacement of ATAAD with dissected distal supra-aortic vessels is intimal inversion, which may subsequently lead to neurological ischemia, prolonged ICU stay, increased reoperation rates and worse clinical outcomes. Advances in endovascular technology have introduced hybrid approaches combining the thoroughness of open surgical repair with the precision of endovascular techniques [4]. Self-expanding covered stents have emerged as a promising solution for managing dissected distal supra-aortic vessels in ATAAD, potentially addressing the limitations of proximal supra-aortic vessel replacement alone.

We report a comparative analysis of self-expanding stent-assisted supra-aortic vessel reconstruction (SAVR) versus proximal replacement of supra-aortic vessels alone (PRVA) in patients who underwent emergency total arch replacement for ATAAD. Our single-center results demonstrate that the SAVR technique could lower the risk of intimal inversion-mediated branch vessel compromise while maintaining equivalent short-term safety profiles.

## 2. Materials and Methods

### 2.1. Study Design and Patient Population

This single-center retrospective comparative study was conducted at Renmin Hospital of Wuhan University between January 2022 and December 2023. The study was conducted in accordance with the Declaration of Helsinki and approved by the Institutional Review Board (WDRY2015 K021), and all patients have provided written informed consent for data utilization. Both the SAVR and PRVA techniques were performed across overlapping calendar time windows during the study period. Group assignment was determined by intraoperative anatomical features of dissected supra-aortic vessels together with senior surgeon clinical judgment, rather than prospective randomization.

**Figure 1:**
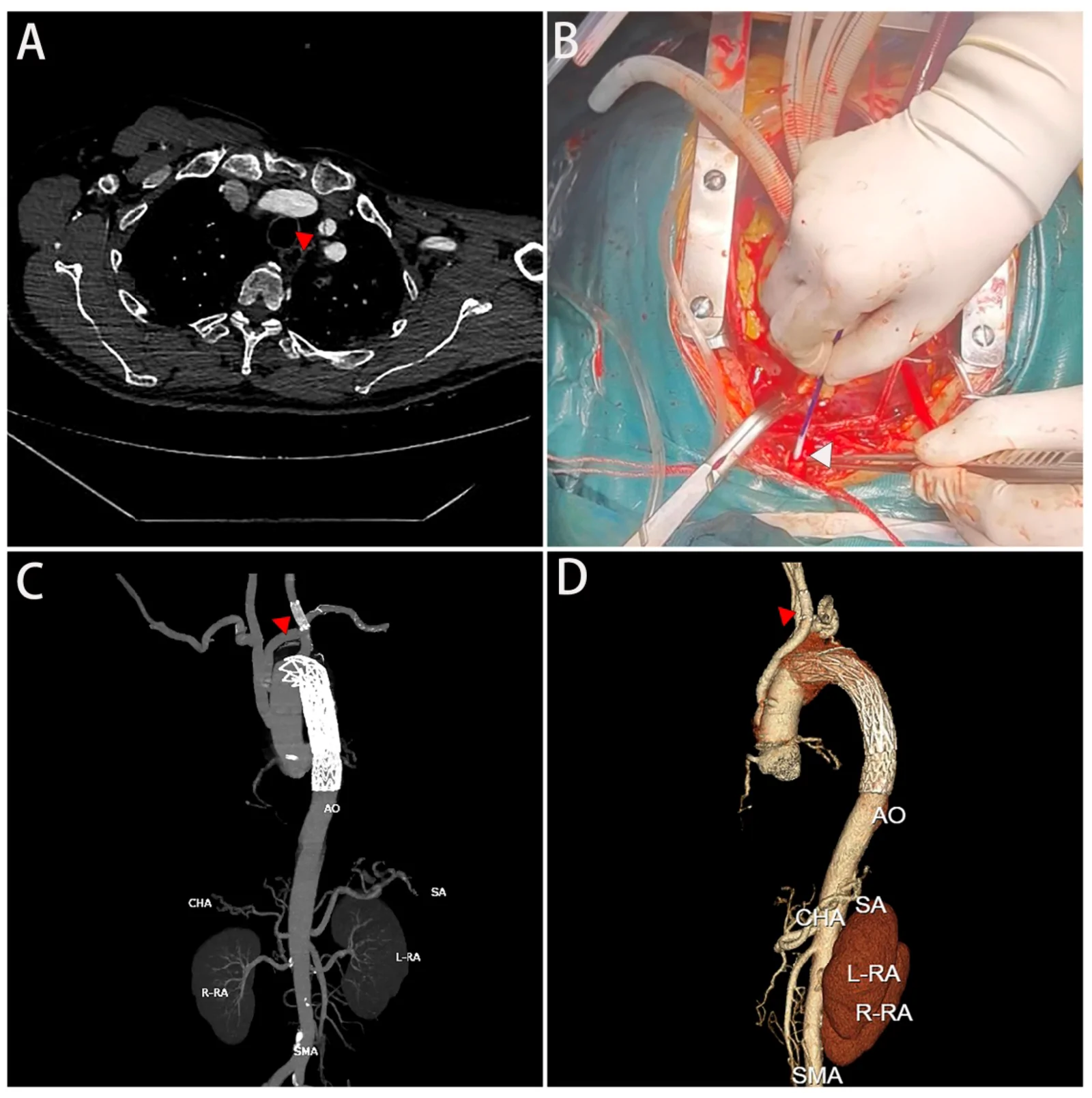
Images of the SAVR technique. (**A**) Dissection extended to distal left carotid artery (red arrow); (**B**) deployment of Viabahn stent into left carotid artery under direct visualization (white arrow); (**C**,**D**) postoperative CTA showed intact branch vessel patency (red arrow). AO: Aorta; SA: Splenic artery; CHA: Common hepatic artery; L-RA: Left renal artery; R-RA: Right renal artery; SMA: Superior mesenteric artery.

Inclusion criteria: (1) acute Stanford type A aortic dissection with patent false lumen; (2) dissection extended into at least one of the supra-aortic vessels (Figure 1A); (3) emergency total aortic arch replacement with the frozen elephant trunk technique was applied; (4) complete perioperative and follow-up data available.

Exclusion criteria: (1) prior cardiac surgery; (2) sub-acute or chronic dissection (>14 days); (3) isolated ascending aortic replacement; (4) contraindications to anticoagulation.

All patients underwent comprehensive preoperative evaluation including transthoracic echocardiography, computed tomography angiography (CTA), and laboratory assessments. Three-dimensional CTA reconstruction enabled precise measurement of vessel diameters and identification of dissection involvement within distal supra-aortic vessels. Device selection for the SAVR group was based on perpendicular diameter measurements at the target landing zones, with minimal oversizing (0–10%) to prevent vessel injury while ensuring adequate seal.

Viabahn covered stents (W.L. Gore & Associates, Flagstaff, AZ, USA) ranging from 8 to 13 mm in diameter were selected based on individual vessel anatomy. Stent length was determined by the extent of dissection involvement, ensuring adequate coverage of the dissected segments while preserving vessel origins. Stents were implanted in the innominate artery, left common carotid artery and/or left subclavian artery as dictated by dissection distribution; each target vessel received one Viabahn stent. The landing strategy required 5–8 mm of healthy arterial segment proximal and distal to the dissected zone for stable stent anchoring.

Postoperative antithrombotic and surveillance protocols were standardized. For SAVR patients receiving Viabahn stents, 100 mg of aspirin was prescribed daily for 12 months postoperatively; therapeutic low-molecular-weight heparin was administered in the early postoperative phase and transitioned to oral aspirin once the bleeding risk was considered acceptable. The PRVA group received antithrombotic therapy according to institutional standard aortic dissection protocols without mandatory long-term single antiplatelet therapy. Surveillance CTA imaging was scheduled at hospital discharge, 1-month, 6-month, and 12-month follow-up, and annually thereafter. Branch vessel restenosis was defined as ≥50% luminal narrowing on reconstructed CTA images. All surviving patients completed this unified follow-up imaging protocol.

### 2.2. Surgical Technique

All procedures were performed under general anesthesia via median sternotomy. Cardiopulmonary bypass was established through femoral or axillary arterial cannulation with right atrial drainage. Moderate hypothermia (28–30 °C) with bilateral antegrade cerebral perfusion at 8 mL/kg∙min as previously described was employed for neuroprotection [5]. Following cooling, the dissected ascending aorta was resected, and a four-branched polytetrafluoroethylene (PTFE) graft was anastomosed to the aortic root. Circulatory arrest was initiated at the target temperature, and the aortic arch was transected at Zone 1. A frozen elephant trunk device (Cronus, MicroPort, Shanghai, China) was deployed antegrade into the true lumen of the descending thoracic aorta, followed by proximal anastomosis to the main PTFE graft. After completion of distal arch repair, systemic circulation was restored, and supra-aortic vessel reconstruction was subsequently performed.

### 2.3. Savr Technique

After restoring systemic circulation, supra-aortic vessel reconstruction commenced. In the SAVR group, pre-selected Viabahn stents were deployed into the dissected supra-aortic vessels under direct surgical visualization (Figure 1B). The stent was positioned to fully overlap the dissected arterial segments. Subsequently, the proximal stent end was anastomosed end to end to the corresponding branch of the four-branched PTFE graft using continuous 5-0 polypropylene suture. This hybrid approach eliminated the requirement for direct manipulation and suturing of the fragile dissected native vessel intima.

### 2.4. Prva Technique

In the PRVA group, conventional end-to-end anastomosis was performed between the proximal portion of the dissected supra-aortic vessels and each PTFE graft branch using continuous 5-0 polypropylene suture. This technique required direct handling and suturing of the dissected native vessel wall, which frequently demanded additional local hemostatic maneuvers.

### 2.5. Data Collection and Outcome Measures

Primary endpoints included: (1) intimal inversion of the supra-aortic vessels and branch vessel patency assessed by CTA at discharge and during the follow-up period (Figure 1C,D); (2) neurological complications including symptomatic and radiographic stroke.

Intimal inversion of a supra-aortic vessel was defined as distal anastomotic segment luminal collapse induced by a retrograde flipped dissection intimal flap, manifesting as luminal obliteration or severe narrowing on postoperative CTA or DSA angiography.

Secondary endpoints included: (1) operative parameters (cardiopulmonary bypass time, circulatory arrest time, cerebral perfusion time); (2) red blood cell transfusion volume; (3) mechanical ventilation time, ICU stay duration; (4) 30-day mortality; (5) reoperation requirements.

Neurological complications were classified as symptomatic (associated with clinical neurological deficit) or radiographic (imaging evidence of cerebral infarction without corresponding clinical symptoms). All imaging studies were reviewed by independent radiologists who were blinded to surgical technique allocation.

### 2.6. Statistical Analysis

Continuous variables are presented as mean ± standard deviation and compared using Student’s *t* test or the Mann–Whitney U test as appropriate. Categorical variables are expressed as frequencies and percentages, analyzed using chi square or Fisher’s exact test. Survival analysis was performed using the Kaplan–Meier methodology with log-rank testing. Statistical significance was defined as *p* < 0.05. All analyses were conducted using Prism 10.6.0 (GraphPad Software, LLC, Boston, MA, USA).

## 3. Results

### 3.1. Baseline Characteristics

Forty-nine patients met the inclusion criteria, with 26 patients (53.1%) in the SAVR group and 23 patients (46.9%) in the PRVA group. The mean age was 51.8 ± 13.4 years, with male predominance (71.4%). Baseline demographics, comorbidities, and anatomical characteristics including the number and distribution of dissected supra-aortic vessels were comparable between groups (Table 1).

Presentation patterns were similar, including malperfusion syndrome (SAVR: 19.2% vs. PRVA: 21.7%, *p* = 1.00), cardiac tamponade (SAVR: 11.5% vs. PRVA: 8.7%, *p* = 1.00), and rupture rate (SAVR: 3.8% vs. PRVA: 4.3%, *p* = 1.00). The distribution location of the entry tear showed no significant differences between groups, with ascending aortic tears being the dominant (SAVR: 53.8% vs. PRVA: 39.1%, *p* = 0.39).

### 3.2. Operative Parameters

Technical success was achieved in all cases with no conversion between techniques. Total operative time was comparable (PRVA: 305 ± 51 vs. SAVR: 327 ± 47 min, *p* = 0.12), despite the additional time required for stent deployment in the SAVR group. Graft-to-branch anastomosis time was slightly longer in the SAVR group (25.8 ± 4.1 vs. 21.3 ± 3.4 min, *p* < 0.01) (Table 2). Cardiopulmonary bypass time (PRVA: 167 ± 29 vs. SAVR: 175 ± 32 min, *p* = 0.35), hypothermic circulatory arrest time (PRVA: 5.1 ± 1.9 vs. SAVR: 5.4 ± 2.1 min, *p* = 0.60), and cerebral perfusion time (PRVA: 26.7 ± 7.8 vs. SAVR: 29.5 ± 8.4 min, *p* = 0.16) were statistically equivalent between groups.

### 3.3. Blood Transfusion

The SAVR group demonstrated significantly reduced transfusion requirements. RBC utilization was lower (SAVR: 4.2 ± 1.7 vs. PRVA: 5.7 ± 1.9 units, *p* < 0.01). Fresh frozen plasma requirements were markedly decreased (SAVR: 308 ± 87 vs. PRVA: 468 ± 71 mL, *p* < 0.01). Platelet transfusion was equivalent between groups (SAVR: 2 vs. PRVA: 2 units, *p* = 1.00) (Table 2).

### 3.4. Supra-Aortic Vessel Patency

The patency of supra-aortic vessels demonstrated significant differences between the two groups. Supra-aortic vessel stenosis occurred in 30.4% (7/23) of PRVA patients secondary to an unopened true lumen; among them, five cases were caused by intimal inversion, and reoperation targeting these compromised vessels was performed. All implanted Viabahn stent bodies remained patent throughout follow-up. One SAVR patient developed branch vessel stenosis located at the proximal anastomotic junction between the PTFE graft branch and stent margin, while the stent segment itself showed no luminal narrowing (Table 3).

### 3.5. Neurological Outcomes

Numerically lower rates of neurological events were observed in the SAVR group, although intergroup differences failed to reach statistical significance. No symptomatic stroke was observed in the SAVR cohort (SAVR: 0% vs. PRVA: 8.7%, *p* = 0.09), and radiographic cerebral infarction was numerically reduced (SAVR: 3.8% vs. PRVA: 13.0%, *p* = 0.17). The single SAVR patient with radiographic cerebral changes experienced transient postoperative encephalopathy with complete recovery. In contrast, posterior circulation strokes among PRVA patients were attributed to supra-aortic vessel compromise secondary to dissection extension.

### 3.6. Recovery Parameters

Mechanical ventilation time (SAVR: 15.2 ± 6.1 vs. PRVA: 21.6 ± 8.4 h, *p* < 0.01) and intensive care unit (ICU) stay (SAVR: 3.1 ± 0.8 vs. PRVA: 4.7 ± 1.1 days, *p* < 0.01) were significantly shorter for patients receiving SAVR. These observed intergroup differences may be influenced by multiple perioperative confounders. Hypothesized explanations, including reduced surgical trauma, are discussed in Section 4.

### 3.7. Safety Outcomes

Thirty-day mortality was comparable between groups (SAVR: 3.8% vs. PRVA: 4.3%, *p* = 1.00). In the SAVR group, one patient died due to multiorgan dysfunction secondary to postoperative sepsis. One death within the PRVA group was attributed to perioperative myocardial infarction. No deaths were directly related to supra-aortic vessel reconstruction.

### 3.8. Follow-Up and Reoperation

The mean follow-up time was 18.2 ± 4.1 months (range: 12–24 months). Reoperation requirements were numerically lower in the SAVR group (SAVR: 3.8% vs. PRVA: 8.7%, *p* = 0.59), though statistical significance was not achieved due to small sample numbers. Two patients in the PRVA group underwent thoracic endovascular aortic repair for distal aortic true lumen remodeling failure. No stent-related complications, including migration, occlusion, or endoleak, were observed during the follow-up period.

## 4. Discussion

This comparative analysis demonstrates that self-expanding stent-assisted supra-aortic vessel reconstruction (SAVR) represents an effective technical modification in acute Stanford type A aortic dissection repair, especially when the dissection extends to distal supra-aortic vessels. The SAVR technique addresses known limitations of proximal vessel replacement of supra-aortic vessels alone (PRVA), achieving superior branch vessel patency while maintaining equivalent short-term safety profiles.

The pathophysiology of dissection-related supra-aortic vessel compromise involves intimal inversion, false lumen propagation, and compromised true lumen perfusion [6]. PRVA requires direct manipulation of fragile dissected vessel walls, which may result in further intimal damage, anastomotic bleeding, and subsequent stenosis. The SAVR approach alters this paradigm by providing immediate true lumen stabilization through radial stent expansion, eliminating the requirement for direct suturing onto diseased native vessel intima.

The self-expanding property of the Viabahn stent offers favorable biomechanical characteristics for dissected supra-aortic vessels. Different from balloon expandable stents, self-expanding devices provide continuous radial force that adapts to vessel remodeling while maintaining luminal patency. The polytetrafluoroethylene covering prevents tissue ingrowth and provides a smooth luminal surface that reduces thrombogenicity [7].

The marked reduction in supra-aortic vessel stenosis within the SAVR group (SAVR: 3.8% vs. PRVA: 30.4%) may contribute to the numerically lower rate of neurological events. Nevertheless, the observed differences in symptomatic and radiographic stroke did not reach statistical significance in this small-sized cohort. Any potential neuroprotective effect remains to be confirmed in larger patient series.

Potential mechanisms for possible neurological benefit extend beyond prevention of stenosis and intimal inversion [8]. By avoiding extensive supra-aortic vessel manipulation, the SAVR technique may reduce the risk of thromboembolism originating from calcified or ulcerated atherosclerotic plaques. Additionally, immediate restoration of true lumen geometry could improve flow dynamics and reduce turbulence-related thrombogenesis [9].

Significantly reduced blood product transfusion, shorter mechanical ventilation and abbreviated ICU stay were noted in the SAVR group. One plausible mechanistic hypothesis is reduced surgical trauma: PRVA often demands extensive tissue exposure and frequent intraoperative hemostatic interventions [10,11,12]. The SAVR approach may minimize such trauma-inducing maneuvers and facilitate hemostasis via stent implantation. These improvements in postoperative recovery could stem from attenuated inflammatory response, reduced coagulopathy, and better-preserved organ function associated with lower blood product exposure [13]. We emphasize, however, that these cross-sectional associations cannot establish definite causal links, as multiple unmeasured perioperative variables may affect transfusion requirements, ventilation duration and ICU length of stay.

Previous studies on aortic arch reconstruction with hybrid approaches have focused primarily on total endovascular approaches or staged procedures [14,15,16]. This study represents the first dedicated comparison of stent-assisted supra-aortic vessel reconstruction performed concomitantly during open repair of acute Stanford type A aortic dissection. The observed outcomes compare favorably with published series of conventional total aortic arch replacement [17,18,19], with particular advantages in preventing branch vessel compromise caused by intimal inversion. The concept of “zone-specific” neuroprotection through targeted supra-aortic vessel management aligns with contemporary understanding of stroke mechanisms in aortic arch surgery [20].

## 5. Limitations and Future Directions

This single-center retrospective study included only 49 patients (26 in the SAVR group, 23 in the PRVA group), representing a relatively small cohort that limits statistical power and generalizability. Patient allocation was determined by intraoperative anatomy and surgeon judgment, which introduces inherent selection and temporal bias even though the baseline clinical and anatomical features were well balanced between groups. Larger-scale multicenter randomized controlled trials are needed to establish definitive clinical superiority across diverse patient populations. Additionally, the mean follow-up period of 18.2 ± 4.1 months is insufficient for comprehensive long-term assessment of stent durability, late complications, and supra-aortic vessel remodeling patterns. Given that acute Stanford type A aortic dissection requires lifelong surveillance, extended observation periods of 5–10 years are essential to fully evaluate the SAVR technique’s safety and efficacy. Finally, while the SAVR approach demonstrates encouraging clinical outcomes, the upfront cost of Viabahn covered stents significantly exceeds that of traditional anastomotic materials. Although the potential cost savings through reduced complications and reoperations may offset initial expenses, formal cost-effectiveness analyses are lacking. Comprehensive health economic evaluations are needed to quantify long-term financial implications and inform evidence-based healthcare decision-making.

## 6. Conclusions

Self-expanding stent-assisted supra-aortic branch reconstruction is a feasible hybrid technical modification for acute Stanford type A aortic dissection repair when dissection involves distal supra-aortic vessels. This technique is associated with improved supra-aortic vessel patency, with numerically favorable trends in neurological and postoperative recovery parameters while maintaining comparable short-term safety. It effectively reduces the occurrence of intimal inversion-related branch vessel compromise. Definitive clinical superiority needs confirmation by larger-sample or multicenter investigations.

## Figures and Tables

**Table 1:** Demographic and anatomical characteristics.

Variable	PRVA (*n* = 23)	SAVR (*n* = 26)	*p* Value
Age (years)	51.4 ± 12.7	52.1 ± 14.2	0.54
Male sex, ***n*** (%)	16 (69.6)	19 (73.0)	1.00
Hypertension, ***n*** (%)	18 (78.3)	23 (88.5)	0.45
Smoking, ***n*** (%)	15 (65.2)	18 (69.2)	1.00
Diabetes mellitus, ***n*** (%)	2 (8.7)	2 (7.7)	1.00
Dyslipidemia, ***n*** (%)	11 (47.8)	10 (43.5)	0.57
COPD, ***n*** (%)	1 (4.3)	2 (7.7)	1.00
Malperfusion, ***n*** (%)	5 (21.7)	5 (19.2)	1.00
Tamponade, ***n*** (%)	2 (8.7)	3 (11.5)	1.00
Rupture, ***n*** (%)	1 (4.3)	1 (3.8)	1.00
Number of dissected supra-aortic vessels	1.7 ± 0.6	1.8 ± 0.7	0.61

Data are expressed as mean ± standard deviation or number (%).

**Table 2:** Operative parameters and blood product utilization.

Variable	PRVA (*n* = 23)	SAVR (*n* = 26)	*p* Value
Total operative time (mins)	305 ± 51	327 ± 47	0.12
Cardiopulmonary bypass time (mins)	167 ± 29	175 ± 32	0.35
Hypothermic circulatory arrest time (mins)	5.1 ± 1.9	5.4 ± 2.1	0.60
Cerebral perfusion time (mins)	26.7 ± 7.8	29.5 ± 8.4	0.16
Graft-to-branch anastomosis (mins)	21.3 ± 3.4	25.8 ± 4.1	<0.01 **
Red blood cell transfusion (units)	5.7 ± 1.9	4.2 ± 1.7	<0.01 **
Fresh frozen plasma transfusion (mL)	468 ± 71	308 ± 87	<0.01 **
Platelet transfusion (units)	2	2	1.00

Data are expressed as mean ± standard deviation. **: Data between groups with statistical difference.

**Table 3:** Clinical outcomes.

Variable	PRVA (*n* = 23)	SAVR (*n* = 26)	*p* Value
Branch vessel stenosis, ***n*** (%)	7 (30.4)	1 (3.8)	<0.01 **
Branch vessel intimal inversion, ***n*** (%)	5 (21.7)	0 (0)	0.02 **
Symptomatic stroke, ***n*** (%)	2 (8.7)	0 (0)	0.09
Radiographic stroke, ***n*** (%)	3 (13.0)	1 (3.8)	0.17
Mechanical ventilation (hours)	21.6 ± 8.4	15.2 ± 6.1	<0.01 **
ICU stay (days)	4.7 ± 1.1	3.1 ± 0.8	<0.01 **
30-day mortality, ***n*** (%)	1 (4.3)	1 (3.8)	1.00
Reoperation, ***n*** (%)	2 (8.7)	1 (3.8)	0.59

Data are expressed as mean ± standard deviation or number (%). **: Data between groups with statistical difference.

## Data Availability

The original contributions presented in this study are included in the article. Further inquiries can be directed to the corresponding authors.

## Abbreviations

The following abbreviations are used in this manuscript:

SAVR	Self-expanding stent-assisted supra-aortic vessel reconstruction
PRVA	Proximal replacement of supra-aortic vessels alone
ATAAD	Acute Stanford type A aortic dissection
CTA	Computed tomography angiography
DSA	Digital subtraction angiography
ICU	Intensive care unit
COPD	Chronic obstructive pulmonary disease
